# Romiplostim in chemotherapy‐induced thrombocytopenia: A review of the literature

**DOI:** 10.1002/cam4.7429

**Published:** 2024-08-12

**Authors:** Gerald A. Soff, Hanny Al‐Samkari, Avi Leader, Melissa Eisen, Hossam Saad

**Affiliations:** ^1^ University of Miami Health System/Sylvester Comprehensive Cancer Center Miami Florida USA; ^2^ Center for Hematology, Massachusetts General Hospital Cancer Center Harvard Medical School Boston Massachusetts USA; ^3^ Section of Hematology, Memorial Sloan Kettering Cancer Center New York New York USA; ^4^ Amgen Inc. Thousand Oaks California USA

**Keywords:** chemotherapy, peptibody, platelet growth factors, romiplostim, thrombocytopenia, thrombopoietin receptor agonists

## Abstract

Chemotherapy‐induced thrombocytopenia (CIT) is a common challenge of cancer therapy and can lead to chemotherapy dose reduction, delay, and/or discontinuation, affecting relative dose intensity, and possibly adversely impacting cancer care. Besides changing anticancer regimens, standard management of CIT has been limited to platelet transfusions and supportive care. Use of the thrombopoietin receptor agonist romiplostim, already approved for use in immune thrombocytopenia, has shown promising signs of efficacy in CIT. In a phase 2 prospective randomized study of solid tumor patients with platelet counts <100 × 10^9^/L for ≥4 weeks due to CIT, weekly romiplostim corrected the platelet count to >100 × 10^9^/L in 93% (14/15) of patients within 3 weeks versus 12.5% (1/8) of untreated patients (*p* < 0.001). Including patients treated with romiplostim in an additional single‐arm cohort, 85% (44/52) of all romiplostim‐treated patients responded with platelet count correction within 3 weeks. Several retrospective studies of CIT have also shown responses to weekly romiplostim, with the largest study finding that poor response to romiplostim was predicted by tumor invasion of the bone marrow (odds ratio, 0.029; 95% CI: 0.0046–0.18; *p* < 0.001), prior pelvic irradiation (odds ratio, 0.078; 95% CI: 0.0062–0.98; *p* = 0.048), and prior temozolomide treatment (odds ratio 0.24; 95% CI: 0.061–0.96; *p* = 0.043). Elsewhere, lower baseline TPO levels were predictive of romiplostim response (*p* = 0.036). No new safety signals have emerged from romiplostim CIT studies. Recent treatment guidelines, including those from the National Comprehensive Cancer Network, now support consideration of romiplostim use in CIT. Data are expected from two ongoing phase 3 romiplostim CIT trials.

## Abstract 2

Chemotherapy‐induced thrombocytopenia (CIT) is a common event in cancer care. Romiplostim has been studied and shown to be an effective therapy for treatment of CIT, although not yet approved by the FDA for this indication. We review the current evidence for romiplostim for CIT.

## 1 INTRODUCTION

Thrombocytopenia can arise from insufficient platelet production, increased platelet destruction, and/or splenic sequestration, representing a range of disorders.^1^, ^2^ Examples include autoimmune disease, bone marrow disorders (e.g., aplastic anemia), and hematologic disorders involving peripheral cytopenias, radiation, surgery, hematopoietic stem cell (HSC) transplantation, or drugs.^3^ Drug‐induced thrombocytopenias account for 20%–25% of all drug‐related hematologic disorders.^4^

Myelosuppression is a common adverse consequence of many anticancer therapies, particularly cytotoxic chemotherapy, and is also found with targeted therapies.^5^, ^6^ The use of supportive care has facilitated more aggressive cancer treatment, aiming to reduce disease progression or achieve remission. Supportive care for chemotherapy‐induced anemia and neutropenia includes red blood cell transfusions, erythropoietin‐stimulating agents, granulocyte colony‐stimulating factors, and antibiotic therapy when indicated. However, until recently, therapy for chemotherapy‐induced thrombocytopenia (CIT) was limited to platelet transfusions and supportive care during severe, symptomatic nadirs; specifically, transfusions to conduct major invasive procedures for platelet counts of ≤50 × 10^9^/L and prophylactic transfusions for <10 × 10^9^/L.^7^ Chemotherapy dose delay or reduction until platelet counts recover is a common strategy for persistent CIT,^6^ often interrupting treatment and possibly impacting outcomes. While CIT incidence varies with cancer type and regimen (solid tumors: 21.9%–64.2%, hematological tumors: 28%–87.2%^6^, ^8^, ^9^), Grades 3–4 CIT is especially notable with hematologic cancers (≤45%, depending on cancer type/regimen) and solid tumors treated with platinum, gemcitabine, or anthracycline chemotherapy (Table 1).^9^

**TABLE 1:** CIT prevalence after chemotherapy by solid tumor type and chemotherapy regimen.^9^

All grades by solid tumor type	
Colorectal	61.7%
Non‐small cell lung	50.5%
Ovarian	45.6%
Breast	37.6%

Grades 3–4 by therapy	
Platinum‐based	10.6%
Gemcitabine‐based	10.6%
Anthracycline‐based	5.2%
Taxane‐based	1.9%

Abbreviation: CIT, chemotherapy‐induced thrombocytopenia.

Thrombocytopenia may be from a number of causes in cancer patients, including bone marrow infiltration, infection, radiation, and hematologic malignancy itself. This manuscript focuses only on management of CIT.

Thrombopoietin (TPO) is the primary growth factor for megakaryocyte proliferation, differentiation, and platelet production.^10^ The TPO receptor (MPL), found on HSCs, megakaryocytes, and platelets, is a homodimeric receptor encoded by the human myeloproliferative leukemia virus (*mpl*) gene.^10^ TPO‐receptor agonists (TPO‐RA) are a class of drugs that bind and activate the TPO receptor but do not contain the peptide sequence of TPO itself. There are currently four available TPO‐RA. Romiplostim is a peptibody.^11^ Eltrombopag, avatrombopag, and lusutrombopag are small molecules.^11^ The current FDA approved indications for TPO‐RA are: Romiplostim (immune thrombocytopenia (ITP) and acute radiation syndrome), eltrombopag (ITP, hepatitis C‐associated thrombocytopenia, severe aplastic anemia), avatrombopag (ITP, periprocedural thrombocytopenia in chronic liver disease), and lusutrombopag (periprocedural thrombocytopenia in chronic liver disease).^11^ There have been multiple studies of TPO‐RA for treatment of CIT^11^, ^12^ To‐date, only studies with romiplostim have been positive.^11^

Here, we provide an overview of CIT and the evidence for use of romiplostim in CIT.

## 2 CHEMOTHERAPY‐INDUCED THROMBOCYTOPENIA

A significant fraction of patients undergoing treatment for solid tumors experience thrombocytopenia.^9^ In addition to classical myelosuppressive chemotherapy, thrombocytopenia may develop with novel agents, including poly (ADP‐ribose) polymerase inhibitors (PARPi), immune checkpoint inhibitors (ICIs), proteasome inhibitors, tyrosine kinase inhibitors (TKIs), histone deacetylase (HDAC) inhibitors, immunomodulatory drugs (IMiDs), and immunotherapies such as chimeric antigen receptor T (CAR‐T) cells.^5^, ^13^, ^14^, ^15^, ^16^, ^17^, ^18^

### 2.1 Mechanisms of CIT

Platelets are produced by megakaryocytes in the bone marrow in response to TPO and consumed through physiologic and pathologic coagulation, apoptosis, and clearance by the spleen and/or liver.^19^, ^20^ Bone marrow suppression leading to thrombocytopenia can be from direct effects on megakaryopoiesis, including platelet release, and/or indirect effects on the bone marrow microenvironment and hematopoietic regulators.^19^, ^22^ Drugs may decrease platelet counts by reducing platelet synthesis, preventing platelet release from megakaryocytes, or increasing platelet apoptosis, destruction, and clearance.^21^

**FIGURE 1:**
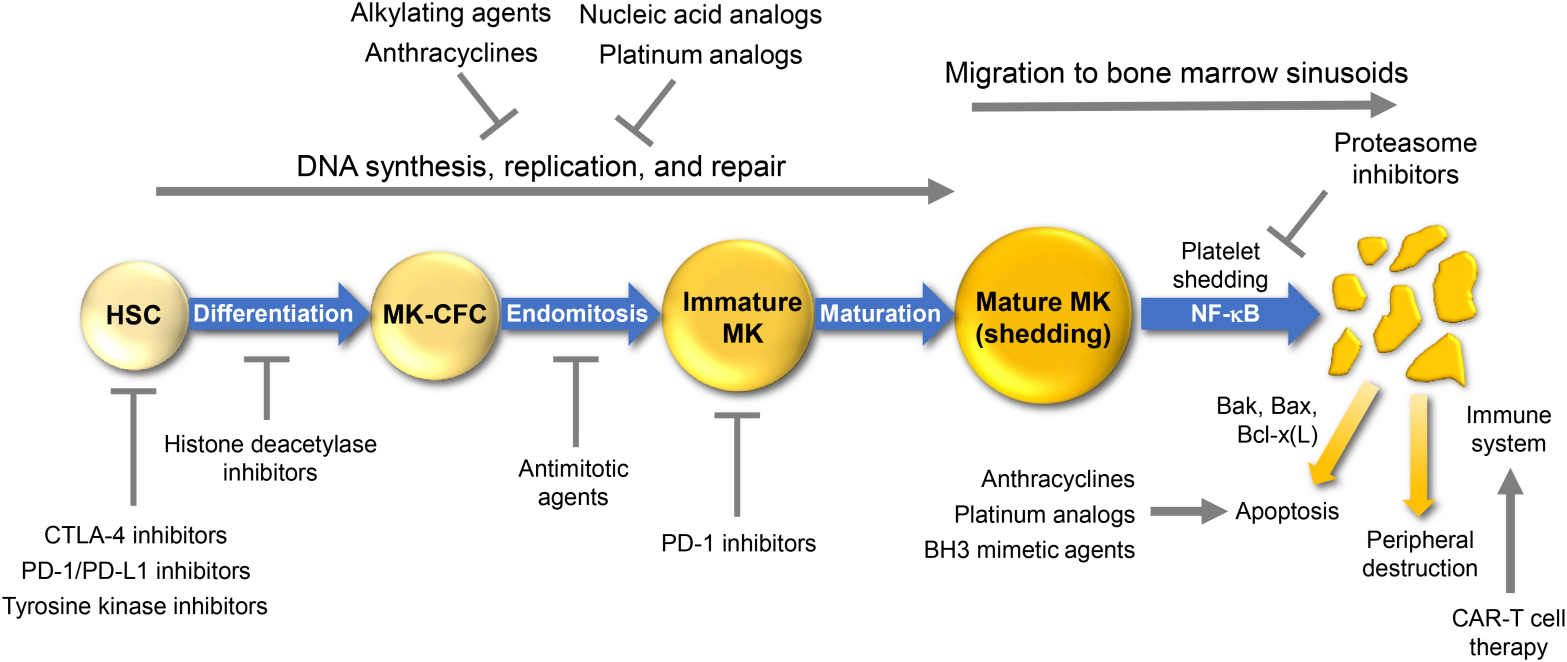
Platelet production and destruction pathways– effect of various anticancer therapies. BH3, Bcl‐2 homology 3; CAR‐T, chimeric antigen receptor T; CTLA, cytotoxic T‐lymphocyte antigen; HSC, hematopoietic stem cell; MK‐CFC, megakaryocyte colony‐forming cell; NF‐κB, nuclear factor kappa B; PD‐1, programmed cell death protein 1; PD‐L1, programmed cell death ligand 1.

While mechanisms by which some anticancer agents lead to thrombocytopenia remain unclear, many pathways have been implicated (Figure 1). These include impairing megakaryocyte and platelet production by affecting DNA synthesis/repair (alkylating agents and platinum analogs),^22^ apoptosis (anthracyclines via Bax, cytochrome c release, and caspase‐3^21^) and the platelet clock Bcl‐x(L) (B‐cell lymphoma 2 [Bcl‐2] homology 3 [BH3] mimetic chemotherapy agent ABT‐737).^1^ Thrombocytopenia may also be mediated through direct effects on HSCs (TKIs)^5^ and cytotoxic T‐lymphocyte antigen‐4 or programmed cell death protein 1 (PD‐1)/PD‐1 ligand (PD‐L1) inhibitors.^23^ Other possible mechanisms include induction of platelet‐specific immunoglobulin G autoantibodies (nivolumab),^17^ lymphodepletion or cytokine release syndrome (CAR‐T cells),^16^ through nuclear factor kappa B (proteasome inhibitors),^24^ and via p53 (HDAC inhibitors).^15^

### 2.2 Incidence of thrombocytopenia with other anticancer therapies

The use of targeted agents can cause thrombocytopenia to varying degrees. Grade 3 or 4 thrombocytopenia was higher with PARPi than conventional chemotherapy (meta‐analysis: 22.8% versus 14.9%, relative risk 1.63, 95% CI: 1.06–2.52, *p =* 0.03).^25^ ICI treatment can result in immune‐related thrombocytopenia in non‐small cell lung cancer (NSCLC),^17^ Hodgkin's lymphoma,^26^ pancreatic cancer,^27^ and renal cell carcinoma.^28^ Further, ITP has been identified as a rare (1.2%) but pertinent cause of treatment‐related death with ICIs. PD‐1 inhibitor monotherapy was associated with a 2% thrombocytopenia rate; PD‐1/PD‐L1 inhibitor with chemotherapy had 6% higher thrombocytopenia risk.^29^

### 2.3 CIT/clinically significant CIT

Although lacking a standard definition, CIT is generally considered to be platelet counts <100 × 10^9^/L persisting despite sufficient time to recover from the prior chemotherapy nadir.^30^, ^31^, ^32^ Specifically, NCCN Guidelines® note that “definitions used in several studies include thrombocytopenia (platelets <100,000/mcL) for ≥3 to 4 weeks following last chemotherapy administration and/or following delays in chemotherapy initiation related to thrombocytopenia.”^33^ The threshold of <100 × 10^9^/L for CIT (vs. <150 × 10^9^/L) reflects when cancer patients are more likely to experience negative consequences from thrombocytopenia (i.e., dose delays, reductions, and discontinuation) and recurrence in future cycles. Key clinical platelet thresholds for *clinically significant CIT* include <50 × 10^9^/L (increased bleeding risk with trauma or surgery),^34^<30 × 10^9^/L (increased ecchymosis/ purpura), and <10 × 10^9^/L, which can necessitate prophylactic platelet transfusion.^7^

The Common Terminology Criteria for Adverse Events (CTCAE) version 5.0 is the standard grading scale for CIT (Table 2).^35^ Platelet nadir depth may worsen with subsequent chemotherapy cycles and increase bleeding risk, making CIT a risk to monitor throughout treatment.^1^ Platelet counts generally start to drop by Day 7, reach a nadir by Day 14, and then gradually return to baseline levels by Days 28–35.^1^ Prolonged thrombocytopenia (≥4 weeks) has been observed in many solid tumor types.^32^ In a retrospective analysis of patients with hematologic or solid malignancy (*n* = 609), of 1262 chemotherapy cycles, 99 (8%) had a chemotherapy dose delay of ≥7 days and 209 (17%) had a chemotherapy dose reduction (i.e., a decrease ≥20% or discontinuation).^36^ Thrombocytopenia contributed to 70% of dose disruptions (delays/reductions) and in ~30% of the 70% cases was the sole cause.^36^

**TABLE 2:** Thrombocytopenia grades and clinical significance.

Grade	Platelet count (×10^9^/L)	Clinical significance
Grade 1	<LLN–75	Mild or asymptomatic; no clinical significance
Grade 2	<75–50	Moderate; not associated with spontaneous bleeding; warrants only minimal intervention if present at start of the next chemotherapy cycle; major surgery should be postponed
Grade 3	<50–25	Severe; patients with vascular disorders and/or coagulopathies may bleed spontaneously, limiting self‐care ADL
Grade 4	<25	Life‐threatening consequences; including risk of spontaneous bleeding or hemorrhagic syndrome; patients with a platelet count of 10–20 × 10^9^/L may have purpura, and platelet transfusion needs must be evaluated

*Note*: Grading per the Common Terminology Criteria for Adverse Events (CTCAE) version 5.0 scale.^35^

Abbreviations: ADL, activities of daily living; LLN, lower limit of normal.

CIT timing also has important implications.^37^ In nadir CIT, platelet counts fall in the middle of the chemotherapy cycle to as low as Grades 3 or 4, but then recover by the beginning of the next cycle. In persistent CIT, platelet counts do not recover adequately for the next scheduled chemotherapy cycle to be given on time at full dose. Alternatively, persistent CIT may be defined as inadequate platelet recovery after chemotherapy is held for 1 week or longer. The efficacy of potential CIT treatments may best be detected with persistent CIT, as nadir CIT can resolve by itself.^38^

### 2.4 CIT‐associated chemotherapy dose delays and reductions

The current CIT management strategy of chemotherapy dose delay, reduction, or discontinuation (Table 3) is not always effective and may adversely impact outcomes.^9^, ^39^, ^40^, ^41^, ^42^, ^43^ Dose delays due to CIT lasting ≥7 days have been seen in patients with advanced NSCLC (32%), breast cancer (33%), and ovarian cancer (44%); likewise, dose reductions of ≥15% were seen in patients with NSCLC (50%), breast cancer (49%), and ovarian cancer (48%).^39^, ^40^ Changes in dosing can be quantified in terms of relative dose intensity (RDI), which is the percentage of delivered chemotherapy dose relative to the reference dose intensity for a specific regimen. Chemotherapy dose reduction or delay are two factors leading to reduced RDI.

**TABLE 3:** Modifications of planned chemotherapy.

Term	Definition
Dose delay	For any given cycle, a ≥7‐day delay from the standard dosing schedule
Dose reduction	A ≥15% decrease in the chemotherapy dose in any given cycle relative to standard dosing
Discontinuation	Stopping chemotherapy altogether

*Note*: These modifications are as described in references.^9^, ^39^, ^40^, ^41^, ^42^, ^43^

Several studies have examined the consequences of reduced RDI. A retrospective study of patients with advanced NSCLC (*N* = 3866) demonstrated a significant association between dose delays of ≥7 days or an RDI <85% and decreased survival, though other causes contributed to decreased RDI.^40^ In a prospective trial in patients with HER2‐negative advanced breast cancer, intermittent administration of chemotherapy was associated with a 38% increased mortality risk.^44^ Further, in a study of metastatic breast cancer, RDI <85% resulted in a significant decrease in overall survival (*p* = 0.0086).^41^ Likewise, in a retrospective study of ovarian cancer, RDI <85% was associated with a 71% increase in mortality.^45^ Overall, a systematic review of RDI in metastatic solid tumors showed that an RDI ≥85% improved progression‐free survival and overall survival.^46^ Thus, while dose modifications may improve platelet counts, alteration of anticancer regimens can worsen survival outcomes for patients.

## 3 OVERVIEW OF CIT MANAGEMENT

The goal of CIT management is to have safe and effective interventions that avoid the need to decrease RDI.^30^, ^36^, ^39^ Preserving RDI remains a key parameter in optimizing patient outcomes; minimizing decreased RDI is thus an important goal when administering chemotherapy.

### 3.1 Earlier‐generation platelet growth factors

Several growth factors were explored but did not change the CIT management paradigm. Oprelvekin (recombinant IL‐11, Neumega®) was approved by the United States Food and Drug Administration to treat CIT, but had modest clinical benefits and significant side effects, leading to it no longer being available.^37^ Initial experience with first‐generation thrombopoietic agents, recombinant human TPO (rhTPO) and pegylated recombinant human megakaryocyte growth and development factor (PEG‐rHuMGDF), was promising for CIT.^1^ However, due to the development of neutralizing antibodies in some patients receiving PEG‐rHuMGDF that cross‐reacted with endogenous TPO and resulted in thrombocytopenia, development of these agents was discontinued. The exception is rhTPO, available in the People's Republic of China for the treatment of ITP and CIT.^37^

### 3.2 Second‐generation platelet growth factors

TPO‐RAs are the second‐generation thrombopoietic agents and a current focus of potential CIT treatments. A systematic literature review and meta‐analysis showed benefit from first‐ and second‐generation TPO‐RAs used to treat or prevent CIT.^47^ The oral TPO‐RA eltrombopag has variable reports of efficacy for CIT.^48^, ^49^, ^50^ Eltrombopag must be taken without a meal or with a meal low in calcium (≤50 mg) and at least 2 h before or 4 h after any medications or products containing polyvalent cations, such as antacids, calcium‐rich food, and mineral supplements,^51^ all of which can be challenging for patients receiving cancer therapy. In a phase 3 trial investigating the orally administered TPO‐RA avatrombopag in patients with solid tumors and primarily nadir CIT, avatrombopag did not meet the composite primary endpoint (avoiding platelet transfusions, chemotherapy dose reductions of ≥15%, and chemotherapy dose delays of ≥4 days) possibly due to the unexpectedly high rates of platelet recovery in the placebo group.^38^ Avatrombopag raised platelet counts and was well tolerated, with a safety profile similar to placebo and no increased venous thromboembolism. These results emphasize the difference between nadir CIT and persistent CIT, and the potential for spontaneous recovery in nadir CIT (which, by definition, does not occur in persistent CIT). Further studies are needed to determine the benefits of thrombopoietic agents for CIT compared with the current management of platelet transfusions or treatment modifications.

## 4 USE OF ROMIPLOSTIM IN THE MANAGEMENT OF CIT

### 4.1 Romiplostim's mechanism of action, pharmacokinetics, and pharmacodynamics

**FIGURE 2:**
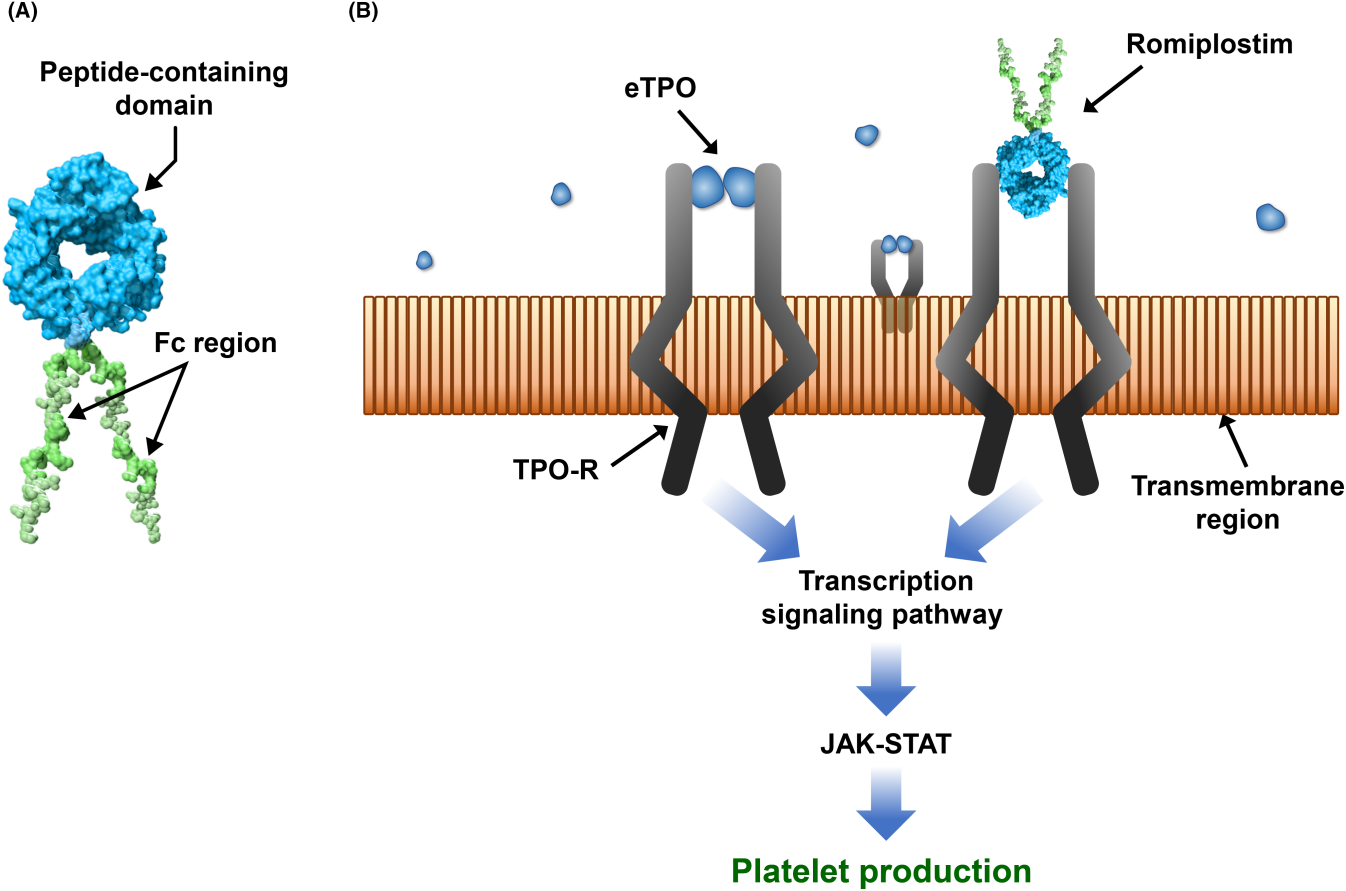
(A) Romiplostim structure. (B) Romiplostim signaling and platelet production. eTPO, endogenous thrombopoietin; JAK, Janus kinase; STAT, signal transducer and activator of transcription; TPO‐R, thrombopoietin receptor. This figure was adapted from a review on romiplostim.^64^

The TPO‐RA romiplostim is a peptibody consisting of four TPO‐receptor binding domains linked to an Fc domain to increase half‐life (Figure 2A) (as reviewed in^3^, ^32^). Romiplostim binding to the TPO receptor results in increased megakaryocyte growth and maturation (Figure 2B). After subcutaneous romiplostim administration, peak serum concentrations occur at a median of 14 h (range: 7–50 h), with a median half‐life of 3.5 days (range: 1–34 days),^52^ and platelet counts increase 4–9 days after administration, peaking on Days 12–16.^53^

### 4.2 Optimal romiplostim dosing in CIT

#### 4.2.1 Evidence on frequency of romiplostim dosing and associated efficacy

While once‐per‐cycle romiplostim dosing did not show effects on platelet counts or chemotherapy dose in lymphoma or NSCLC,^53^, ^54^ multiple romiplostim CIT studies found weekly dosing important in maximizing efficacy (Table SS1). In a phase 2 randomized study of patients with solid tumors with platelet counts <100 × 10^9^/L for at least 4 weeks due to CIT, weekly romiplostim corrected the platelet count (≥100 × 10^9^/L) in 93% (14/15) of patients within 3 weeks as compared with 12.5% (1/8) of untreated patients (*p* < 0.001).^32^ Including patients treated with romiplostim in an additional single‐arm cohort, 85% (44/52) of all romiplostim‐treated patients responded with platelet count correction within 3 weeks. Likewise, a comprehensive retrospective study of 173 patients with CIT (153 had solid tumors, 20 lymphomas or myelomas) showed that weekly romiplostim dosing was superior to intermittent intracycle dosing, with higher median platelet counts (143 × 10^9^/L vs. 106 × 10^9^/L, *p* < 0.001) and response rates (81% vs. 63%, *p* = 0.006) and correspondingly fewer chemotherapy dose reductions/delays (82 vs. 224 per 100 patient‐years, *p* = 0.010) and fewer bleeding events (11 vs. 34 per 100 patient‐years, *p* = 0.029).^30^ The same study showed a numerical reduction in venous thromboembolism with weekly dosing (7.1 vs. 20 per 100 patient‐years, *p* = 0.25).^30^ In a retrospective analysis of patients with solid tumors, 59% (10/17) of patients with baseline platelet counts <100 × 10^9^/L achieved platelet counts ≥100 × 10^9^/L with a single romiplostim dose, with six more reaching that threshold after 2–12 weekly doses.^55^ Another retrospective analysis of weekly romiplostim found that platelet counts improved in all patients, reaching ≥100 × 10^9^/L in 19/20 patients.^56^ Lastly, weekly romiplostim was effective in facilitating weekly maintenance therapy with temozolomide in patients with glioblastoma.^57^ In all, studies in both solid and hematologic cancers support weekly romiplostim dosing in CIT.

#### 4.2.2 Evidence on romiplostim dose selection, titration, and timing

In studies in which romiplostim showed clinical benefit,^30^, ^32^, ^55^, ^56^, ^57^, ^58^ weekly weight‐based dosing was adjusted by platelet count. Typically, the target platelet count was 100 × 10^9^/L (often also the measure of response) as that allowed chemotherapy to resume and surgery to proceed as necessary.^30^, ^32^, ^55^, ^56^ If platelet counts rose above 200 or 400 × 10^9^/L, a dose titration schema was specified to hold or decrease the dose accordingly. The starting dose was generally 1–3 mcg/kg, with an escalation of 1 mcg/kg each week. Specifically, in the phase 2 prospective randomized study,^32^ dosing was initiated at 2 mcg/kg and increased by 1 mcg/kg for up to 3 weeks to target platelet counts ≥100 × 10^9^/L, with the mean doses required to achieve and maintain target platelet counts being 2.6 mcg/kg and 3.3 mcg/kg, respectively. In follow‐up of those receiving ≥1 year of romiplostim, the mean dose remained ~3–5 mcg/kg up to 3 years.^58^ Retrospective studies showed similar patterns, with dosing typically initiated at 1–3 mcg/kg and adjusted to target platelet counts ≥100 × 10^9^/L; the resulting optimized dose was ~3 mcg/kg for both solid and hematological cancers.^30^, ^34^, ^55^, ^56^, ^59^, ^60^ Based on both animal and clinical studies, administering romiplostim on the same day as chemotherapy is associated with comparable outcomes as administration on other days of the chemotherapy cycle.^3^, ^30^, ^32^, ^59^, ^60^

### 4.3 Clinical outcomes with romiplostim in CIT, response and safety

Available data indicate that romiplostim corrects CIT in most patients and that maintenance romiplostim allows for chemotherapy resumption and continuation without CIT recurrence (Table SS1). In the phase 2 study of patients with solid tumors, 85% (44/52) of patients resumed chemotherapy with maintenance romiplostim after CIT correction; only 6.8% of patients (3/44) experienced recurrent chemotherapy dose reduction/delay due to isolated CIT.^32^ When patients receiving romiplostim maintenance and chemotherapy were followed‐up for ≥1 additional year, 70% (14/20) of patients had no further CIT, four had a single dose delay, and two required dose reductions.^58^ The mean romiplostim dose remained stable at 3–5 mcg/kg up to 3 years, indicating no loss of effect. 10% (2/20) of patients had thrombotic events (one a deep vein thrombosis, one multiple tumor‐related infarctions), an incidence expected for patients with metastatic disease. There was no clinical evidence of either bone marrow fibrosis or secondary hematologic malignancies. The cancer associated thrombosis rate was not increased compared with historical evidence in patients with metastatic cancer, undergoing chemotherapy.^30^, ^32^, ^58^ Overall, data from this study indicate that romiplostim shows promising efficacy without new safety concerns when given in either the short or long term to patients with CIT.^32^, ^58^

Retrospective studies have also reported promising data for romiplostim in CIT (Supplemental Table S1 **)**. The aforementioned study of 173 patients receiving weekly or intracycle romiplostim dosing had overall findings of significantly raised median platelet counts vs. baseline (116 × 10^9^/L vs. 60 × 10^9^/L, *p* < 0.001) and a response rate of 71%. Further, bleeding rates were lesser than reported historically in similar CIT populations and venous thromboembolic events occurrence was as expected for this patient population.^30^ The vast majority of patients were able to avoid chemotherapy dose reductions/delays (79%) and platelet transfusions (89%). This study expanded upon earlier smaller retrospective analyses of CIT in romiplostim‐treated patients with various solid tumor types (*n* = 20).^56^

The large number of patients in the weekly/intracycle study allowed for multivariable logistic modeling of response to romiplostim in patients receiving various regimens (i.e., platins, taxanes, gemcitabine, fluorouracil, irinotecan, and temozolomide). Model results indicated that poor response to romiplostim could be predicted by tumor invasion of the bone marrow (odds ratio, 0.029, 95% CI: 0.0046–0.18, *p* < 0.001), prior pelvic irradiation (odds ratio, 0.078, 95% CI: 0.0062–0.98, *p* = 0.048), and prior temozolomide treatment (odds ratio, 0.24, 95% CI: 0.061–0.96, *p* = 0.043).^30^ Patients with these predictors of romiplostim non‐response had considerably lower platelet counts and response rates of 23%, 20%, and 46%, respectively. Further, in an observational cohort study of 63 patients with CIT receiving romiplostim,^61^ 54/63 (86%) patients achieved a response (platelet count ≥75 × 10^9^/L and ≥30 × 10^9^/Lower baseline), with higher predicted response associated with lower baseline TPO levels (*p* = 0.036 in a generalized linear model). TPO levels <100 pg/mL predicted responses at ≥60% of platelet assessments in >90% of cases, whereas TPO levels >2000 pg/mL likewise predicted a lack of these responses in >90% of cases.

### 4.4 CIT management guidelines

Current NCCN Guidelines for Hematopoietic Growth Factors indicate that possible use of romiplostim is a level 2A recommendation.^33^ The guidelines continue “The primary purpose of using TPO‐RAs for CIT is to maintain dose schedule and intensity of chemotherapy when such benefit is thought to outweigh the potential risks.”^33^ Further, the European Society for Medical Oncology supportive care guidelines in COVID note that “Thrombopoietin mimetics should be considered in patients with severe thrombocytopenia after chemotherapy.”^62^ For romiplostim, the dosing strategy per National Comprehensive Cancer Network® (NCCN®) “includes weekly dosing beginning at 2–4 mcg/kg, increased no more than 1–2 mcg/kg per week, to target platelet count of 100,000–150,000/mcL.”^33^, ^63^ Of note, per the prescribing information, the maximum dose of romiplostim for ITP is 10 mcg/kg weekly.^52^ The NCCN Guidelines further describe data for avatrombopag, eltrombopag, and lusutrombopag and state that “Insufficient data are available to support use of TPO‐RAs other than romiplostim for CIT outside of a clinical trial.”^33^ Regarding the phase 3 trial of avatrombopag in 122 patients with solid tumors and severe CIT, while avatrombopag‐treated patients did have somewhat higher platelet counts and treatment was deemed safe, this study did not meet its primary endpoint (a composite of avoiding platelet transfusions, chemotherapy dose reductions of ≥15%, and chemotherapy dose delays by ≥4 days).^38^

Current International Society of Thrombosis and Hemostasis guidelines recommend participation in a clinical trial for CIT, but add, “If unable to enroll in a clinical trial, we suggest consideration of a TPO‐RA in the setting of inadequate platelet recovery at day 1 of a chemotherapy cycle to avoid chemotherapy dose reduction or a delay of ≥7 day assuming adequate neutrophil and hemoglobin recovery”.^11^

## 5 FUTURE DIRECTIONS

There currently is no consensus definition for CIT, as platelet count thresholds typically vary from 50 to 100 × 10^9^/L. There also is no standard nomenclature to describe the timing and persistence of thrombocytopenia. Establishing uniform terminology will help distinguish clinically relevant conditions, such as nadir vs. persistent CIT, which have clinical consequences. For clinicians wanting to treat CIT with romiplostim, predicting which patients would most benefit and refining the goal of therapy are of particular interest. There are data indicating that tumor invasion of the bone marrow, prior pelvic irradiation, prior temozolomide treatment, and higher baseline TPO levels are all predictors of non‐response to romiplostim, however, prediction of response to romiplostim remains subject to ongoing study. The existing evidence indicates that the use of romiplostim in patients with CIT improves the ability to maintain full RDI of cancer therapy; however, whether cancer control is improved is yet to be demonstrated. These knowledge gaps should be considered in future clinical research and study design.

## 6 CONCLUSIONS

Currently, there are limited options and no medical consensus regarding how to treat CIT to decrease bleeding and platelet transfusions and allow for optimal chemotherapy dosing. The development of the TPO‐RAs as a possible treatment option has renewed interest in managing CIT, particularly with data expected soon from many trials for multiple agents, including phase 3 trials of romiplostim (NCT03362177 and NCT03937154).

## AUTHOR CONTRIBUTIONS

**Gerald A. Soff:** Conceptualization (equal); formal analysis (equal); investigation (equal); methodology (equal); writing – original draft (equal); writing – review and editing (equal). **Hanny Al‐Samkari:** Writing – original draft (equal); writing – review and editing (equal). **Avi Leader:** Writing – original draft (equal); writing – review and editing (equal). **Melissa Eisen:** Writing – original draft (equal); writing – review and editing (equal). **Hossam Saad:** Writing – original draft (equal); writing – review and editing (equal).

## CONFLICT OF INTEREST STATEMENT

G.A.S.: Consultant for Amgen, Janssen, Bayer Pharmaceuticals, Sobi, Bristol‐Myers Squibb, Pfizer, Novartis, Anthos Therapeutics; research funding from Amgen, Janssen, Sobi. H.A‐S consultant for Agios, Alnylam, Alpine, Amgen, Sobi, Argenx, Novartis, Pharmacosmos; research funding from Agios, Sobi, Amgen, Novartis, Vaderis. A.L.: consultation/advisory board fees from Bayer, Novartis, Pfizer, Sanofi. M.E., H.S.: Amgen employees and stockholders.

## Supporting information


Table S1.

## Data Availability

None.
