# Supplementary material for: Romiplostim in chemotherapy‐induced thrombocytopenia: A review of the literature
Source: Cancer Med. 2024 Aug 12;13(15):e7429. doi: 10.1002/cam4.7429 (PMC11319220; doi:10.1002/cam4.7429)
Supplement: Supplementary file 1 — Table S1. [file CAM4-13-e7429-s001.docx]

## Supplemental Table 1: Studies of romiplostim in patients with CIT

| **Study type** | **Eligibility** | **Patients (N)** | **Dose of romiplostim** | **Efficacy** | **Adverse events** |
| --- | --- | --- | --- | --- | --- |
| Phase 1/2 – dose-finding study in lymphoma ^1^ | Adults with histologically confirmed Hodgkin lymphoma or NHL treated with Q14-, Q21-, Q28-day CHOP, ICE, ESHAP, or DHAP ± rituximab, who experienced platelet count ,50×10^9^/L (grade 3 or 4 thrombocytopenia) in a previous chemotherapy cycle using the same regimen and doses. Also required were adequate bone marrow function on the start day of next on-study chemotherapy cycle and ANC ≥1×10^9^/L, hemoglobin ≥9.5 g/dL, platelet count >100×10^9^/L. Excluded were those with more than one prior chemotherapy regimen, history of thromboembolic disease, or prior positive test for hepatitis B or C, or HIV infection. | 39 | Cohort 1 (n=8) 100 µg SC on day 1  Cohort 2 (n=11) 300 µg SC on day 1  Cohort 3 (n=11) 700 µg SC on day 1  Cohort 4 (n=9) 1000 µg SC on day 1 | There was no evidence that once-per-cycle romiplostim improved the platelet nadir or other efficacy endpoints. | AEs typical for lymphoma patients receiving chemotherapy. No dose-dependent effects were observed. |
| Phase 2 – randomized, placebo-controlled, dose-finding study in NSCLC ^2^ | Adults with stage IIIB or IV NSCLC treated with Q21-day gemcitabine + platinum chemotherapy who had thrombocytopenia (platelet count ,100×10^9^/L) in a previous cycle | 63 | Cohort 1 (n=16) 250 µg SC on day 2  Cohort 2 (n=18) 500 µg SC on day 2  Cohort 3 (n=16) 750 µg SC on day 2  Placebo (n=12) | There was no evidence that once-per-cycle romiplostim improved platelet count–related efficacy endpoints or chemotherapy dose reduction. A caveat is that there was an imbalance in the chemotherapy regimens between the placebo and romiplostim groups, with more romiplostim patients receiving the carboplatin-containing regimen. | Romiplostim treatment was well tolerated. AE rates were similar for romiplostim and placebo groups; the incidence of serious AEs was numerically higher with romiplostim without any dose-dependent effects. No neutralizing antibodies to romiplostim or thrombopoietin were identified. |
| Phase 1/2 – randomized, placebo-controlled, dose-finding study in NHL^3^ | Adults with NHL receiving treatment with R-hyper-CVAD or R-Arac/MTX in cycle 1 | 50 | Cohort 1 (n=12)  1 μg/kg SC or placebo 2:1  Cohort 2 (n=12)  3 μg/kg SC or placebo 2:1  Cohort 3 (n=12)  10 μg/kg SC or placebo 2:1  Cohort 4 (n=12)  Placebo  Arm A: Before and after chemotherapy dosing on  days –5 and 5  Arm B: After chemotherapy dosing on days 5 and 7  Up to six cycles | Romiplostim given both before and after chemotherapy (Arm A) significantly increased the platelet nadir and decreased thrombocytopenia duration. There were also fewer patients with bleeding events (all grades) and platelet transfusions in those treated with romiplostim before and after chemotherapy (Arm A). | Romiplostim was generally well tolerated. VTE occurred in both romiplostim-treated (4/41) and placebo-treated (4/9) patients on study. |
| Phase 1/2 – dose-finding study in patients with solid tumors^4^ | Adults with solid tumors receiving carboplatin, adriamycin + ifosfamide, or high-dose ifosfamide | 24 | Cohort 1 (n=6)  1 μg/kg SC  post-chemotherapy dosing days 1 and 3  Cohort 2 (n=6)  3 μg/kg SC  post-chemotherapy dosing days 1 and 3  Cohort 3 (n=6)  10 μg/kg SC  post-chemotherapy dosing days 1 and 3  Cohort 4 (n=10)  10 μg/kg SC  pre- and post-chemotherapy dosing days –5 and 1  Up to six cycles | Romiplostim showed efficacy using pre- and post-dosing in patients receiving carboplatin and stabilized or increased platelet counts in cycle 2 vs. cycle 1 | Romiplostim was generally well tolerated |
| Phase 2 – open-label, randomized study of romiplostim vs. observation in locally advanced or metastatic solid tumors^5^, follow-up for those with ≥1 year of romiplostim ^6^ | Adults with locally advanced or metastatic solid tumors with CIT (platelets <100×10^9^/L) for  ≥4 weeks, despite a chemotherapy dose delay or reduction with ECOG PS 0–2 and no chemotherapy for 14 days prior to enrollment. Patients were previously treated with carboplatin or cisplatin, an anthracycline, an alkylating agent, a nucleoside analog, or other cytotoxic agent known to cause thrombocytopenia. | 60 | Initiated at 2 µg/kg, increased by 1 µg/kg for ≤3 weeks, adjusted to achieve a platelet count of  ≥100×10^9^/L | Platelet counts ≥100×10^9^/L were achieved within 3 weeks by 93% of patients treated with romiplostim vs. 12.5% of observed patients. Overall, platelet correction was achieved in 85% of patients receiving romiplostim (44/52); 64% of these patients (28/44) resumed the same chemotherapy as before. Among patients on romiplostim for ≥1 year, 14/20 had no further CIT, four had a single dose delay, and two had dose reductions. The mean dose remained ~3–5 µg/kg up to 3 years. | VTE was seen in 10% (6/59) of the patients in the first year, romiplostim was continued in these patients. Among patients on romiplostim for ≥1 year, there was one DVT and one patient with multiple tumor infarctions. There was no clinical evidence of either bone marrow fibrosis or secondary hematologic malignancies. |
| TMZ-induced thrombocytopenia in glioblastoma:  the PLATUM trial^7^ | Adults with histologically confirmed newly diagnosed glioblastoma, with ECOG PS 0–2, and the intent to treat set with standard first-line concomitant and maintenance TMZ with radiotherapy over 6 weeks, and CTCAE grade 3 or 4 thrombocytopenia. Eligible patients had adequate bone marrow function, ANC ≥1×10^9^/L, hemoglobin ≥9.5 g/dL, and platelet count >100×10^9^/L before initiation of TMZ. Excluded were those with coagulopathy or hematologic disease including thrombocytopenia before initiation of TMZ. | 20 | Cohort 1 (n=16)  Romiplostim given during or within 4 weeks after  concomitant radiotherapy/TMZ: weekly romiplostim for 3 weeks, 7 days after radiotherapy/ TMZ  Cohort 2 (n=4) Romiplostim given during maintenance TMZ  Up to six cycles | 60% of the patients with TMZ-induced grade 3 or 4 thrombocytopenia were able to complete six cycles of maintenance TMZ. Romiplostim did not negatively affect progression-free survival and overall survival. | No major bleeding or severe AEs related to romiplostim |
| Retrospective case series of solid tumors^8^ | Patients with persistent thrombocytopenia (platelet count <100×10^9^/L for ≥6 weeks) despite chemotherapy dose delay or reduction and no bone marrow disorder or other etiology for thrombocytopenia. Romiplostim was not offered to patients with cytogenetics associated with leukemia or myelodysplastic syndrome | 20 | Romiplostim initiated at 1–2 μg/kg, increased by 1 μg/kg weekly until platelet counts recovered | Weekly romiplostim dosing raised platelet counts, allowing for chemotherapy to resume for most patients | Thrombosis rate (15%) was within the expected range for this population; three patients with DVT were treated with enoxaparin and stayed on study with no further thromboses. There was no evidence of bone marrow abnormalities due to romiplostim. |
| Retrospective case series^9^ | Adults with solid tumors who received concurrent romiplostim and chemotherapy | 42 received ≥1 dose (safety set), 37 received ≥1 doses (efficacy set) | Varied, typically once or twice between chemotherapy cycles with a median of 2 weeks between doses and a median starting dose of 2 µg/kg | 35/37 patients reached platelet counts >100×10^9^/L, 34/37 received at least 2 more chemotherapy cycles, patients received a median of five chemotherapy cycles | Thrombosis in 6/42 patients (five pancreatic cancer, one astrocytoma), none fatal, not associated with platelet count or romiplostim dose |
| Retrospective analysis of solid tumors^10^ | Adults who received romiplostim concurrently with cancer therapy for a solid tumor; those who received <2 romiplostim doses were excluded. | 22 | ≥2 sequential weekly doses of romiplostim, usually administered on the same day as chemotherapy | It took a median of 7 days of romiplostim (median starting dose 3 μg/kg) to reach ≥75×10^9^/L or ≥100×10^9^/L platelet counts. All patients received ≥2 chemotherapy cycles while on romiplostim. Chemotherapy delays and reductions were significantly reduced in frequency and duration. | No thrombotic AEs observed; three patients experienced bleeding events. Platelet transfusions were received by four patients and red blood cell transfusions were received by 10 patients (eight for chemotherapy-associated anemia or fatigue). |
| Multicenter study of solid  and hematologic malignancies^11^ | Adults with persistent thrombocytopenia (platelet count <100×10^9^/L ≥3 weeks) from last chemotherapy or delay of ≥1 week in starting chemotherapy because of thrombocytopenia | 173 | Weekly or intracycle dosing per institutional guidelines until the end of chemotherapy | Romiplostim improved platelet counts, decreased chemotherapy dose reduction/delays, bleeding, and platelet transfusions; 98% of the patients continued chemotherapy with romiplostim support. Compared with intracycle dosing, weekly dosing showed improved outcomes and fewer CIT recurrences. Romiplostim was not effective in those with tumor invasion in bone marrow or history of prior pelvic irradiation or prior exposure to TMZ. | Not applicable for this modeling study |
| Observational cohort study^12^ | Adult patients with CIT who received romiplostim and had baseline TPO levels measured; data abstracted from the Mass General Brigham Research Patient Data Registry. | 63 | Weekly romiplostim institutional dosing pathway; most patients were started at 2 or 3 µg/kg/week and titrated by 1–2 µg/kg/week | Overall, 54/63 (86%) patients achieved a response (platelet count ≥75×10^9^/L and ≥30×10^9^/L over baseline), with responding a greater fraction of the time associated with lower baseline TPO levels (*p*=0.036 in a generalized linear model). | Not applicable for this modeling study |

AE, adverse event; ANC, absolute neutrophil count; CHOP, cyclophosphamide, doxorubicin, vincristine, prednisolone; CIT, chemotherapy-induced thrombocytopenia; CTCAE, Common Terminology Criteria for Adverse Events; DHAP, dexamethasone AraC cisplatin; DVT, deep vein thrombosis; ECOG PS, Eastern Cooperative Oncology Group Performance Status; ESHAP, etoposide, methylprednisolone, cytarabine, cisplatin; HIV, human immunodeficiency virus; ICE, ifosfamide, carboplatin, etoposide; MTX, methotrexate; NHL, non-Hodgkin lymphoma; NSCLC, non-small cell lung cancer; R‑AraC, rituximab, cytarabine; R-hyper-CVAD, rituximab, cyclophosphamide, vincristine sulfate, doxorubicin hydrochloride (Adriamycin), and dexamethasone; SC, subcutaneous; TMZ, temozolomide; TPO, thrombopoietin; VTE, venous thromboembolism.

# References

1. ClinicalTrials.gov. A Dose and Schedule Finding Trial With AMG 531 for Chemotherapy Induced Thrombocytopenia (CIT) in Adults With Lymphoma. [www.clinicaltrials.gov/NCT00283439.Accessed](https://cactusglobal-my.sharepoint.com/personal/utkarsha_singh_cactusglobal_com/Documents/AMGEN_UT/Npl-MAN-011589-CIT%20review/May%202023/www.clinicaltrials.gov/NCT00283439.Accessed) September 28, 2021.

2. Natale R., Charu V., Schutte W. ea. Safety of romiplostim for treatment of chemotherapy-induced thrombocytopenia (CIT) in patients with advanced non-small cell lung cancer (NSCLC). Eur J Cancer. 2009;7: 574.

3. Vadhan-Raj S, Hagemeister F, Fayad LE, et al. Randomized, double-blind, placebo-controlled, dose and schedule-finding study of AMG 531 in chemotherapy-induced thrombocytopenia (CIT): Results of a phase I/II study. Blood. 2010;116: 1544.

4. ClinicalTrials.gov. AMG 531 in Patients With Advanced Malignancy Receiving Treatment With Carboplatin. <https://www.clinicaltrials.gov/ct2/show/NCT00147225.Accessed> February 28, 2023.

5. Soff GA, Miao Y, Bendheim G, et al. Romiplostim Treatment of Chemotherapy-Induced Thrombocytopenia. J Clin Oncol. 2019;37: 2892-2898.

6. Wilkins CR, Ortiz J, Gilbert LJ, et al. Romiplostim for chemotherapy-induced thrombocytopenia: Efficacy and safety of extended use. Res Pract Thromb Haemost. 2022;6 (3): e12701.

7. Le Rhun E, Devos P, Houillier C, et al. Romiplostim for temozolomide-induced thrombocytopenia in glioblastoma: The PLATUM trial. Neurology. 2019;93: e1799-e1806.

8. Parameswaran R, Lunning M, Mantha S, et al. Romiplostim for management of chemotherapy-induced thrombocytopenia. Support Care Cancer. 2014;22: 1217-1222.

9. Miao J, Leblebjian H, Scullion B, Parnes A. A single center experience with romiplostim for the management of chemotherapy-induced thrombocytopenia. Am J Hematol. 2018;93: E86-e88.

10. Al-Samkari H, Marshall AL, Goodarzi K, Kuter DJ. The use of romiplostim in treating chemotherapy-induced thrombocytopenia in patients with solid tumors. Haematologica. 2018;103: e169-e172.

11. Al-Samkari H, Parnes AD, Goodarzi K, Weitzman JI, Connors JM, Kuter DJ. A multicenter study of romiplostim for chemotherapy-induced thrombocytopenia in solid tumors and hematologic malignancies. Haematologica. 2021;106: 1148-1157.

12. Song AB, Goodarzi K, Karp Leaf R, Kuter DJ, Al-Samkari H. Thrombopoietin level predicts response to treatment with romiplostim in chemotherapy-induced thrombocytopenia. Am J Hematol. 2021;96: 1563-1568.
